# Human Pleural Fluid and Human Serum Albumin Modulate the Behavior of a Hypervirulent and Multidrug-Resistant (MDR) *Acinetobacter baumannii* Representative Strain

**DOI:** 10.3390/pathogens10040471

**Published:** 2021-04-13

**Authors:** Camila Pimentel, Casin Le, Marisel R. Tuttobene, Tomas Subils, Jasmine Martinez, Rodrigo Sieira, Krisztina M. Papp-Wallace, Niroshika Keppetipola, Robert A. Bonomo, Luis A. Actis, Marcelo E. Tolmasky, Maria Soledad Ramirez

**Affiliations:** 1 Center for Applied Biotechnology Studies, Department of Biological Science, College of Natural Sciences and Mathematics, California State University Fullerton, Fullerton, CA 92831-3599, USA; camilapimentel99@csu.fullerton.edu (C.P.); thanhle1998@csu.fullerton.edu (C.L.); mtuttobene13@gmail.com (M.R.T.); jm13070@usc.edu (J.M.); mtolmasky@fullerton.edu (M.E.T.); 2 Instituto de Procesos Biotecnológicos y Químicos de Rosario (IPROBYQ, CONICET-UNR), Rosario S2002LRK, Argentina; tomassubils@gmail.com; 3 Fundación Instituto Leloir—IIBBA CONICET, Buenos Aires C1405BWE, Argentina; rsieira@leloir.org.ar; 4 Research Service and GRECC, Louis Stokes Cleveland Department of Veterans Affairs Medical Center, Cleveland, OH 44106, USA; krisztina.papp@va.gov (K.M.P.-W.); Robert.Bonomo@va.gov (R.A.B.); 5 Departments of Medicine, Pharmacology, Molecular Biology and Microbiology, Biochemistry, Proteomics and Bioinformatics, Case Western Reserve University School of Medicine, Cleveland, OH 44106, USA; 6 CWRU-Cleveland VAMC Center for Antimicrobial Resistance and Epidemiology (Case VA CARES), Cleveland, OH 44106, USA; 7 Department of Chemistry and Biochemistry, California State University Fullerton, Fullerton, CA 92831-3599, USA; nkeppetipola@Fullerton.edu; 8 Department of Microbiology, Miami University, Oxford, OH 45056, USA; actisla@miamioh.edu

**Keywords:** huma serum albumin, *Acinetobacter baumannii*, quorum sensing, iron, human fluids

## Abstract

*Acinetobacter baumannii* is a nosocomial pathogen capable of causing serious infections associated with high rates of morbidity and mortality. Due to its antimicrobial drug resistance profile, *A. baumannii* is categorized as an urgent priority pathogen by the Centers for Disease Control and Prevention in the United States and a priority group 1 critical microorganism by the World Health Organization. Understanding how *A. baumannii* adapts to different host environments may provide critical insights into strategically targeting this pathogen with novel antimicrobial and biological therapeutics. Exposure to human fluids was previously shown to alter the gene expression profile of a highly drug-susceptible *A. baumannii* strain A118 leading to persistence and survival of this pathogen. Herein, we explore the impact of human pleural fluid (HPF) and human serum albumin (HSA) on the gene expression profile of a highly multi-drug-resistant strain of *A. baumannii* AB5075. Differential expression was observed for ~30 genes, whose products are involved in quorum sensing, quorum quenching, iron acquisition, fatty acid metabolism, biofilm formation, secretion systems, and type IV pilus formation. Phenotypic and further transcriptomic analysis using quantitative RT-PCR confirmed RNA-seq data and demonstrated a distinctive role of HSA as the molecule involved in *A. baumannii*’s response.

## 1. Introduction

*Acinetobacter baumannii*, a nosocomial and community acquired pathogen frequently resistant to multiple drugs, causes a wide variety of infections associated with high mortality rates [1,2]. Highlighting the importance of *A. baumannii*, the CDC’s 2019 Antibiotic Resistance Threats Report listed this pathogen among the urgent threat category [3]. *A. baumannii*’s intrinsic antibiotic resistance, metabolic versatility, resistance to different stressors, and high genomic plasticity are responsible for its high adaptability [4,5,6]. A key feature of *A. baumannii*’s adaptability is its capacity to change its metabolism and nutritional needs [7,8]. Additionally, bacterial sensing of host environmental signals has been proposed to play a critical role in these adaptation processes. 

Studies examining the differential expression of specific genes of *A. baumannii* when exposed to different bodily fluids showed that *A. baumannii* could respond to these stimuli by shaping its pathogenic behavior [8,9,10,11,12,13]. Recent findings showed that when exposed to a variety of sterile human fluids, *A. baumannii* A118 displays large-scale complex responses affecting phenotypes highly relevant to bacterial persistence and infection [8,9,10]. For instance, human pleural fluid (HPF), a medium that primarily functions to lubricate pleurae during respiratory movements, triggers in *A. baumannii* a transcriptional response affecting a large number of genes related to metabolic processes [8]. Moreover, we observed that purified human serum albumin (HSA), which is the most abundant blood protein and an important component of HPF, indeed triggers a larger transcriptional response in *A. baumannii* A118, not only affecting natural transformation-related gene expression but also motility, efflux pumps, pathogenicity, and antibiotic resistance, among others [10,14,15]. Additionally, we noted that strains with diverse degrees of pathogenicity respond differently to modifications of external conditions. For example, the mildly pathogenic strain A118 responds with larger transcriptional and phenotypic changes than other more pathogenic strains, when exposed to human fluids [8,9]. Supporting this observation, transcriptomic analysis performed for A118 and AB5075 strains exposed to cerebrospinal fluid (CSF) revealed that AB5075 did not show any significant changes at the transcriptomic level. In contrast, A118 displayed differences in its transcriptome [15].

In the present work, we extend our previous observations and verify the distinctive and decreased transcriptional response of the hypervirulent and extremely drug resistant strain AB5075 [16] exposed to HSA or HPF. This transcriptional analysis revealed a surprising response that was centered on genes associated with quorum sensing, fatty acid metabolism, motility, transport, uptake, and iron storage, among others. 

## 2. Results and Discussion

### 2.1. Hypervirulent and MDR A. baumannii Transcriptome Response to Human Fluids

The transcriptomic analysis of *A. baumannii* strain AB5075 revealed that HPF and HSA significantly affect the expression of 31 and 30 coding genes, respectively (FDR adjusted *p*-value of <0.05 and log_2_ fold change >1). Eleven and 12 of these genes were up-regulated in the presence of HPF or HSA, respectively. The expression levels of 11 genes were modified in the presence of either fluid, with nine and two of them being up- and down-regulated, respectively. Genes whose expression was regulated by the presence of HPF or HSA encoded functions related to quorum sensing, fatty acid metabolism, motility, bacterial survival, efflux pump, and biofilm, and functions associated with transport, uptake, and iron storage (Table S1). Nine out of the 11 genes responsive to the presence of HPF or HSA coded for functions related to quorum sensing (four) and fatty acid metabolism (five) (Table S1).

The number of *A. baumannii* AB5075 genes whose expression is modified by HPF or HSA was surprisingly low compared to HPF’s effect on the mildly virulent strain *A. baumannii* A118, in which case more than 1120 genes were differentially expressed thus it experienced a higher responsiveness to external stimuli [8,9]. The molecular and physiological bases behind these differences remain to be determined.

### 2.2. Human Fluids Enhance the Expression of Genes Involved in Quorum Sensing and Quorum Quenching in a Hypervirulent and MDR A. baumannii Strain

**Figure 1:**
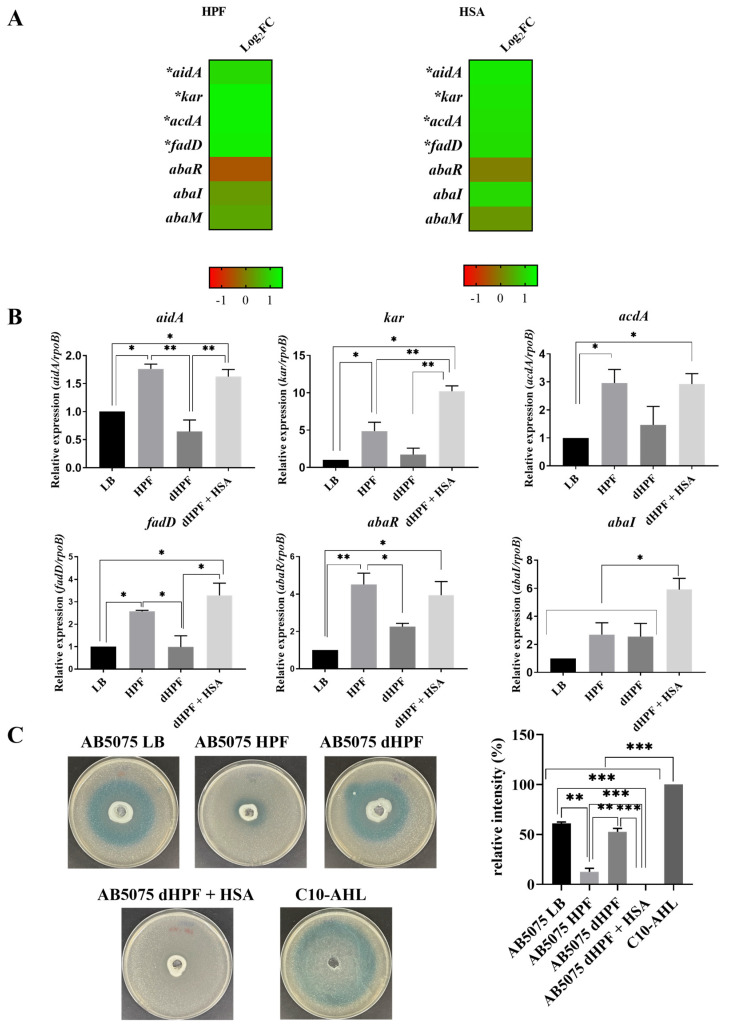
Phenotypic and genetic analysis of quorum sensing coding genes. (**A**) Heatmap outlining the differential expression of genes associated with quorum sensing in presence of HPF or HSA. The majority of quorum sensing associated genes are up-regulated (green) in the presence of HPF and HSA. The asterisks represent the differentially expressed genes (DEGs) (adjusted *p* < 0.05 with log2fold change >1), one asterisks: *p* < 0.05; two asterisks: *p* < 0.01 and three asterisks: *p* < 0.001. (**B**) qRT-PCR of AB5075 strain genes associated with quorum sensing, *aidA, kar, acdA, fadD, abaR* and *abaI* expressed in LB or LB supplemented with HPF, dHPF, or dHPF + HSA. Fold changes were calculated using double ΔCt analysis. At least three independent samples were used. LB was used as the reference condition. (**C**) Agar plate assay for the detection of AHL using *A. tumefaciens*. The presence of AHL were determined by the development of the blue color. Quantification of 5,5’-dibromo-4,4’-dichloro-indigo were estimated as the percentage relative to C10-AHL standard, measured with ImageJ (NIH). The mean ± SD is informed. Statistical significance (*p* < 0.05) was determined by ANOVA followed by Tukey’s multiple-comparison test.

Analysis of mRNA extracted from *A. baumannii* AB5075 cells cultured in the presence or absence of HPF or HSA showed that the expression of four out of seven genes associated with quorum sensing and quorum quenching was significantly enhanced (Table S1 and Figure 1A). The *kar*, *acdA*, and *fadD* genes code for 3-oxoacyl-ACP reductase, Acyl-CoA dehydrogenase, and Acyl CoA synthase, respectively. All three enzymes participate in AHLs synthesis [17]. The *aidA* gene codes for the quorum quenching α/β hydrolase AidA, which catalyzes the hydrolysis of the auto-inducer that results in inhibition of the motility and biofilm formation in *A. baumannii* [17]. 

To verify the role of HSA in promoting changes in levels of expression of quorum sensing related genes, we carried out quantitative RT-PCR (qRT-PCR) assays using total RNA extracted from *A. baumannii* AB5075 cells cultured in LB or LB supplemented with either HPF, HSA-depleted HPF (dHPF), or dHPF supplemented with HSA (dHPF + HSA) (Figure 1B). All four genes, *aidA*, *kar*, *acdA*, and *fadD*, were up-regulated by up to 5-fold and 10-fold in medium supplemented with HPF or dHPF + HSA, respectively. Modifications in expression levels were not observed when dHPF was added to the medium. These results strongly suggest that the HSA component of HPF is the molecule responsible for inducing the differential expression of these genes. qRT-PCR experiments showed that expression of *abaR* and *abaI* (not listed as DEGs, Table S1) is enhanced in cells cultured in medium supplemented with HSA (Figure 1B).

To determine if the differential expression of the genes highlighted above is correlated with phenotypic modifications in quorum sensing, we assessed the levels of acyl homoserine lactone (AHL) using the *Agrobacterium tumefaciens*-based solid plate assays [18]. The *A. baumannii* supernatants from cultures using medium containing dHPF or LB produced a blue color of high intensity, indicative of the presence of large chain AHLs. Conversely, the supernatants from cultures containing HPF or dHPF + HSA produced low intensity or undetectable color, likely caused by increased lactonase activity (quorum quenching) or reduced amounts of large chain AHLs (Figure 1C). These results suggest that (a) there is large chain AHLs-mediated communication between *A. baumannii* AB5075 cells and (b) the large chain AHLs can be degraded, or their production inhibited by components present in HPF, such as HSA (Figure 1). The assays using culture medium supplemented with dHPF + HSA suggest that the component responsible for the low concentrations of large chain AHLs is HSA. Taken together, the results generated by RNA-seq data, qRT-PCR analysis, and the quorum sensing phenotypic assays, we conclude that upon sensing the presence of HSA, *A. baumannii* AB5075 triggers a quorum quenching response. Determination of levels of short chain AHLs, carried out using the biosensor *Chromobacterium violaceum* based solid plate assays [19], showed halos of violacein were not evident (Figure S1). This was an expected result because it is known that the most predominant *A. baumannii* AHL is 3-hydroxy-C12-homoserine lactone [20,21,22]. 

Notably, while carrying out the *C. violaceum*-based solid plate assays, we observed a growth inhibition halo around the supernatant spot. This growth inhibitory effect was observed with supernatants from *A. baumannii* AB5075 cultures supplemented with HPF. Distinctive, yet smaller halos were also observed around locations where supernatants from cultures supplemented with dHPF or dHPF + HSA were spotted. An effect was not observed when testing cultures in LB without any supplement (Figure S1). This bacteriocin-like effect could render *A. baumannii* AB5075 a competitive advantage compared to other bacteria in the lung niche [23]. A direct analysis of the *A. baumannii’s* secretome when growing in different conditions will be exceptionally informative. These experiments will help us to identify the mechanisms and molecule(s) responsible for this growth inhibitory effect.

### 2.3. Iron Acquisition Is Modulated in Presence of Human Fluids in the Hypervirulent, MDR A. baumannii Strain AB5075

As it is the case with numerous pathogens [24,25,26,27], previous research showed that the action of high-affinity iron-uptake systems contribute to *A. baumannii* pathobiology and virulence [28]. Upon entry into the human host, *A. baumannii* encounters extremely low concentrations of free iron as a consequence of the nutritional immunity [29,30,31]. The human host responds to the invasion of the pathogen reducing the already low free-iron levels in blood and tissue fluids triggering a set of reactions called the hypoferraemic response [24,32,33]. To survive, and thrive, under these conditions of iron starvation, like other bacterial pathogens, *A. baumannii* has developed several iron acquisition strategies, such as the production of different siderophores which are variably produced in different strains and likely account for Fe (III) scavenging from different sources [34]. 

**Figure 2:**
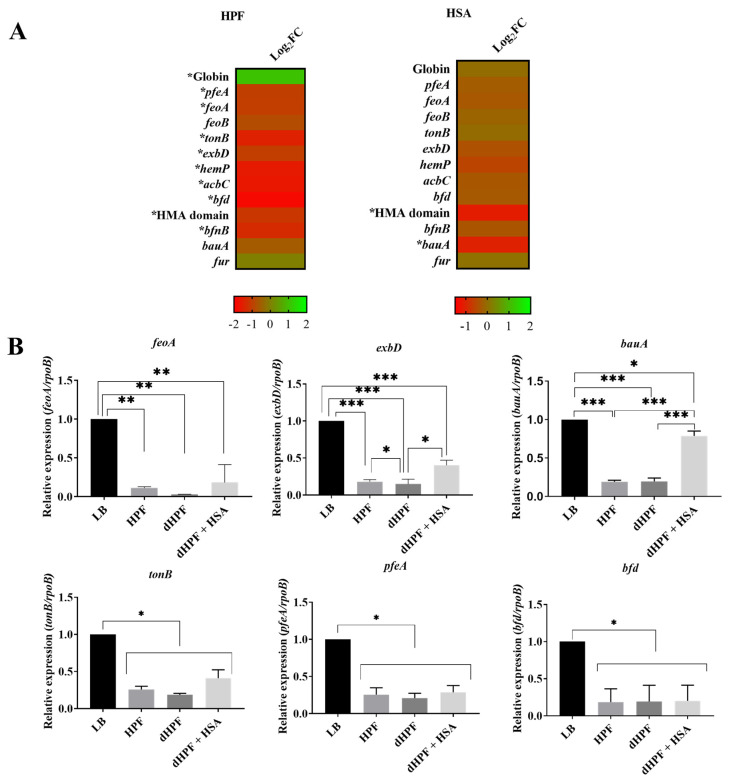
Phenotypic and genetic analysis of iron uptake genes. (**A**) Heatmap outlining the differential expression of genes (DEGs) associated with iron uptake in presence of HPF or HSA. The majority of iron uptake associated genes are down-regulated (red) in the presence of HPF and HSA. The asterisks represent the DEGs (adjusted *p*-value < 0.05 with log2fold change >1). (**B**) qRT-PCR of AB5075 strain genes associated with iron uptake, *feoA, exbD, bauA, tonB, pfeA* and *bfd* expressed in LB or LB supplemented with HPF, dHPF, or dHPF + HSA. Fold changes were calculated using double ΔCt analysis. At least three independent samples were used, and four technical replicates were performed from each sample. The LB condition was used as reference. Statistical significance (*p* < 0.05) was determined by ANOVA followed by Tukey’s multiple-comparison test, one asterisks: *p* < 0.05; two asterisks: *p* < 0.01 and three asterisks: *p* < 0.001.

Transcriptomic analysis of *A. baumannii* AB5075 showed that expression of nine genes related with iron acquisition and metabolic functions was significantly reduced in the presence of HPF (Figure 2A). The genes most inhibited by the presence of HPF were *feoA, bfnB, bfd, acbC, hemP, tonB, pfeA,* HMA and *exbD* induced the expression of globin gene (Figure 2A). The *feoA* gene belongs to the ferrous iron transport cluster (*feoABC*) [35] and proteins encoded by *bfnB* and *acbC* participate in biosynthesis of baumannoferrin, a hydroxamate siderophore [11,36]. The *tonB, exbD* and *pfeA* genes code for inner membrane protein complexes implicated in the transport of ferric-siderophores from the extracellular milieu to the cytosol [37]. The gene product of *hemP* is involved in the uptake of the iron source hemin [38] and *bfd* encodes a bacterioferritin-associated ferredoxin, a key component of the iron homeostasis machinery [39]. The HMA domain (heavy-metal-associated domain) is a conserved protein domain found in a number of heavy metal transport proteins [40]. The expression levels of *feoA* and *exbD* were confirmed to be statistically significant by qRT-PCR analysis (Figure 2B). Both genes were reduced approximately 0.2- and 0.4-fold when the cells were incubated in media supplemented with HPF or dHPF + HSA, respectively. A more significant reduction of the expression of *bauA*, which codes for the outer membrane receptor of ferric-acinetobactin complexes [41], and the gene coding for the heavy-metal-associated domain (HMA domain) protein was detected when the culture medium was supplemented with HSA (Figure 2A). 

The regulation of expression of *bauA* in different growth conditions was also determined by qRT-PCR. The results of these experiments show a statistically significant down-regulation in *bauA* expression in the presence of HPF or dHPF + HSA (Figure 2B). The total Fe concentration in HPF is about 130 μM while that of LB, dHPF and dHPF + HSA is 89 μM, 99 μM, and 67 μM, respectively (as determined by a colorimetric iron assay kit, Sigma-Aldrich, MO, USA) (Figure S2 and Table S2). These results support that the inhibition of the expression of 14 of the 17 coding genes belonging to the *bas*-*bau* gene cluster (Figure S3), a group of genes required for expression of the acinetobactin-mediated iron acquisition system [42], by the addition of HPF to LB could be due to the higher iron content of this host fluid because of the presence of significant concentrations of the iron chelating protein ferritin [43]. On the other hand, the increased expression of the *bas*-*bau* gene in the presence of HSA reflects the lower iron content of LB supplemented with dHPF or dHPF + HSA (Table S2). Furthermore, the expression of *tonB, pfeA* and *bfd* analyzed by qRT-PCR demonstrated statistically significant down-regulation in the presence of HPF, dHPF, or dHPF + HSA (Figure 2B). Taken together, these observations reveal that, as it is the case with most pathogens, *A. baumannii* senses and responds to the lack of free iron in human fluids adjusting the expression of genes coding for components of iron-uptake mechanisms such as the acinetobactin and baumannoferrin mediated systems.

### 2.4. Human Fluids Shift Fatty Acid Metabolic Gene Expression in A. baumannii AB5075

*A. baumannii* undergoes changes in its metabolism and nutritional needs under unfavorable conditions [7,8]. Genome-scale modeling of *A. baumannii* AB5075 in a murine infection model showed significant metabolic changes that help the bacteria adapt to the host during bacteremia. The metabolic changes previously observed were associated with the tricarboxylic acids cycle, gluconeogenesis, nucleotide and amino acid metabolism, as well as with biosynthesis of various cell components like peptidoglycan, lipopolysaccharide, and fatty acids [44]. Furthermore, recent evidence in *A. baumannii* links the fatty acid β-oxidation pathway with protection against host long-chain polyunsaturated fatty acids [45]. These fatty acids are not commonly found in the membranes of bacterial pathogens and are known to have antibacterial activity [45]. Three genes code for lipid biosynthetic functions. The *phaC*, which codes for a protein implicated in the synthesis of poly 3-hydroxyalkanoic acid [46], was significantly up-regulated in HPF and HSA conditions; the *pgaC*, which codes for a product implicated in synthesis of cell-associated poly-β-(1-6)-N-acetylglucosamine (PNAG) [47], was down-regulated in both conditions, but statistically significantly only in HSA condition; and the fatty acid (FA) hydroxylase gene, which is responsible of the hydroxylation of the carboxyl end of fatty acids [48,49], was statistically significantly down-regulated in HPF and close to zero in HSA condition (Figure S4A). The *scd* gene codes for an stearoyl-CoA 9-desaturase, enzyme used to produce the monounsaturated fatty acid oleic acid from the saturated fatty acid stearic acid. The presence of HPF or HSA showed that the expression of two *sdc* genes was significantly enhanced (Figure S4A). The differential expression of *fadA, phaC, and scd* was further confirmed to be up-regulated by qRT-PCR analysis, which revealed up to 2-fold increase in expression in medium containing HPF and dHPF + HSA for *fadA and phaC,* and 2-fold increase in expression in medium containing dHPF + HSA for *scd* (Figure S4B). Taken together, these results support the role of HSA in modulating gene expression and a possible role in changes in fatty acid metabolism associated with the survival of *A. baumannii* within the host. These changes might compensate the deficiency or limited availability of components needed for survival of the bacterial cells.

### 2.5. The Catabolism of Acetoin Is Altered in the Presence of Human Fluids in A. baumannii AB5075

Acetoin (3-hydroxy-2-butanone) is an important molecule that prevents over-acidification of the cytoplasm as well as the surrounding environment resulting from the accumulation of acidic metabolic products [50,51]. The presence of HPF or HSA in the growth medium did not result in a significant reduction in the expression of eight genes associated with the acetoin/butanediol catabolic pathway genes (Figure S5A). However, qRT-PCR experiments revealed a statistically significant decrease in the expression of *acoA* in cells growing in medium supplemented with dHPF (0.113-fold, *p*-value: 0.0420). The same assays showed no difference in expression of *acoB* (Figure S5B). Growth curves performed in M9 minimal medium containing 15 mM acetoin inoculated with overnight culture of AB5075 previously grown in LB, dHPF, HPF, or dHPF + HSA showed that of all four conditions *A. baumannii* AB5075’s growth was most deficient in the presence of dHPF (Figure S5C). Growth in minimal medium containing acetoin supplemented with overnight culture of AB5075 grown in HPF or dHPF + HSA or LB gave no statistically significant differences (Figure S5C). The data shown in this section are consistent with a role in growth modulation by acetoin when in combination with some human fluids.

### 2.6. Human Fluids Affect the Expression of Others Important Genes 

We found genes associated with antibiotic resistance that displayed significantly modified expression (Figure S6A). An efflux pump-related gene was up-regulated in the presence of HPF or HSA. Conversely, the fluoroquinolone tolerance gene *dnpA* [52] and the colistin resistance gene *pmrA* [53], were down-regulated by the presence of HSA (Figure S6A). Genes associated with resistance to β-lactams were not modified by addition of HPF or HSA (Figure S7). Other genes down-regulated in the presence of HPF were *wrbA*, *pspC and motA*. The *wrbA* and *pspC* genes code for stress regulators [54,55], and *motA* is a gene essential for motility [56] (Figure S6A). Addition of HSA was associated with reduced expression of *katE* and *ywrO,* which code for a catalase and a general stress protein, respectively [57]. A gene belonging to the Hly-III family, which codes for cytolysis associated functions [58], was up-regulated by HSA (Figure S6A). HSA also induced down-regulation of *csuB*, a component of the *csu* operon which is essential for *A. baumannii* biofilm formation [59] (Figure S6A). Measurements carried out by qRT-PCR confirmed the reduced expression of *csuB* in media containing HPF, dHPF, or dHPF + HSA. The variations in expression levels of *csuB* were 0.115-fold (*p* < 0.0001), 0.863-fold (*p* = 0.0041) and 0.164-fold (*p* <0.0001), in HPF, dHPF or dHPF+HSA, respectively (Figure S6B). The production of biofilm, a critical element for survival and persistence on abiotic surfaces and pathogenicity, was reduced when *A. baumannii* AB5075 cells were cultured in LB with addition of HPF, dHPF, or dHPF + HSA (Figure S6C). 

## 3. Materials and Methods

### 3.1. Bacterial Strains

*A*. *baumannii* strain AB5075 was used as the model system. It is known to be highly virulent and resistant to antimicrobials [16,54].

### 3.2. RNA Extraction, Sequencing, and Transcriptomic Analysis

RNA extraction was done as previously described [10]. Briefly, AB5075 cells were cultured in Luria Bertani broth (LB) with or without 0.2% HSA or 4% HPF and incubated with agitation for 18 h at 37 °C. Overnight cultures were then diluted 1:10 in fresh LB broth and incubated with agitation for 7 h at 37 °C. RNA was immediately extracted following the TRI REAGENT® Kit (Molecular Research Center, Inc., Cincinnati, OH, USA) as previously described [10]. HPF was obtained from a certified vendor (Innovative Research Inc, Novi, MI, USA ) and pure HSA was obtained from Sigma-Aldrich, St. Louis, MO, USA. 

RNA sequencing was outsourced to Novegene (Novogene Corporation, Sacramento, CA, USA), where the RNA-seq library preparation (Illumina, San Diego, CA, USA) and HiSeq 2500 paired-end 150 bp sequencing of three independent biological replicates in the presence or absence of HSA or HPF was performed. Trimming of low-quality bases at the ends of the reads to a minimum length of 100 bp and removal of Illumina adaptor sequences was performed using Trimmomatic [60], yielding an average of 7.6 million paired reads per sample. FastQC (www.bioinformatics.babraham.ac.uk/projects/fastqc/) (January 2019) was used to assess the quality of the reads before and after trimming. Burrows–Wheeler Alignment software (BWA) was used to align the RNA-seq reads to the genome of *Acinetobacter baumannii* AB5075 [61]. The alignments were visualized using the Integrated Genome Viewer software [62]. FeatureCounts [63] was used to calculate the read counts per gene, and differential expression analysis was performed using DEseq [64]. Features exhibiting FDR <0.05 and log_2_fold change >1 were considered statistically significant. Both RNA-seq data were deposited in the Gene Expression Omnibus (GEO) database under the accession number GSE167117.

### 3.3. HSA Depletion

To obtain HSA-depleted HPF (dHPF), 1 mL of HPF was placed into a 3 kDa Amicon™ Ultra Centrifugal Filter (Millipore, Temecula, CA, USA) and the solution was centrifuged at 20,000× *g* for 10 min. To verify HSA depletion, an SDS-PAGE was conducted that contained 4% HPF and dHPF (Figure S8). 

### 3.4. Quantitative Reverse Transcription Polymerase Chain Reaction (qRT-PCR)

RNA extracted and DNase-treated from *A. baumannii* strain AB5075 grown in LB and LB supplemented with 4% HPF, 4% dHPF, or 4% dHPF + 0.2% HSA, was used to synthesized cDNA using the manufacturer protocol provided within the iScript^TM^ Reverse Transcription Supermix for qPCR (Bio-Rad, Hercules, CA, USA). The cDNA concentrations were adjusted to a concentration of 50 ng/μl. qPCR was conducted using the iQ^TM^ SYBR^®^Green Supermix through the manufacturer’s instructions. At least three biological replicates of cDNA were used and were run in quadruplet. All samples were then run on the CFX96 Touch^TM^ Real-Time PCR Detection System (Bio-Rad, Hercules, CA, USA).

The transcript levels of each sample were normalized to the *rpoB* transcript levels for each cDNA sample. The relative quantification of gene expression was performed using the comparative threshold method 2^−ΔΔCt^. The ratios obtained after normalization were expressed as folds of change compared with cDNA samples isolated from bacteria cultures on LB. Significance differences were determined by ANOVA followed by Tukey’s multiple comparison test (*p* < 0.05), using GraphPad Prism (GraphPad software, San Diego, CA, USA).

### 3.5. N-Acyl Homoserine Lactone (AHL) Detection

*Agrobacterium tumefaciens*-based solid plate assays were carried out to detect AHL production [65]. Briefly, 500 µL of the homogenate were loaded in a central well of 0.7% LB agar plates supplemented with 40 μg of 5-bromo-3- indolyl-b-D-galactopyranoside (X-Gal) per mL and 250 µL (OD = 2.5) of overnight cultures of *Agrobacterium tumefaciens* biosensor. The presence of AHL was determined by the development of the blue color [18,65]. As a positive control, 100 µL of N-Decanoyl-DL-homoserine lactone (C10-AHL) 12.5 mg/mL was utilized. Quantification of 5,5’-dibromo-4,4’-dichloro-indigo production in different conditions was determined by measuring the intensity of each complete plate, and substracting the intensity measured in the negative control, using ImageJ software (NIH). The values were normalized to the positive control, which received the arbitrary value of 100. Furthermore, *Chromobacterium violaceum*-based solid plate assays [19] were also carried out to detect short chain AHL. Briefly, 500 µL of culture supernatants of AB5075 cultured in LB or LB supplemented with 4% HPF, 4% dHPF, or 4% dHPF + 0.2% HSA, were loaded in wells made in LB plates overlaid with 5 mL of a 1/100 dilution of an overnight culture of *C. violaceum* CV026 in soft agar (0.8%). Plates were incubated for 24 h at 30 °C and the production of violacein was examined.

### 3.6. Growth in the Presence of Acetoin

To test the ability of *A. baumannii* AB5075 to grow on acetoin as the sole carbon source, 1/50 dilutions of overnight cultures grown in LB, 4% HPF, 4% dHPF, and 4% dHPF + 0.2% HSA were inoculated in LB or LB plus 10 mM or 15 mM acetoin for 15 hours at 37 °C with medium shaking. Growth was measured at an OD_600_ every 20 min using a Synergy 2 multi-mode plate reader (BioTek, Winooski, VT, USA) and Gen5 microplate reader software (BioTek).

### 3.7. Biofilm Assays

Biofilms assays were performed as previously described [10]. AB5075 cells were cultured in LB or LB supplemented with 4% HPF, 4% dHPF, or 4% dHPF + 0.2% HSA with agitation for 18 h at 37 °C. Experiments were performed in triplicate, with at least three technical replicates per biological replicate.

### 3.8. Determination of Total iron Concentration

Iron Assay Kit (Sigma-Aldrich) were used following the manufacturer’s recommendations, to test the total iron concentration of the different media used in this work, LB or LB supplemented with 4% HPF, 4% dHPF, or 4% dHPF + 0.2% HSA.

### 3.9. Statistical Analysis 

All experiments were performed at least in technical and biological triplicate. Data were expressed as means ± standard deviation. Statistical analysis using Mann–Whitney test or ANOVA followed by Tukey’s multiple comparison test were performed using GraphPad Prism (GraphPad software, San Diego, CA, USA), and a *p* value < 0.05 was considered statistically significant.

## 4. Conclusions

The data described here in this article support our previous observations in *A. baumannii* A118, a clinical isolate susceptible to most antibiotics and less virulent than the AB5075 strain [8,9,10,14,66]. These results indicate that *A. baumannii* strains may sense the HSA in human fluids and respond regulating expression critical genes. 

A limited number of genes (31 and 30 HPF- and HSA-regulated genes, respectively), were modulated in the MDR and hypervirulent strain *A. baumannii* AB5075 compared to the susceptible strain A118 (1120 and 296 HPF- and HSA-regulated genes, respectively), reinforcing the concept of strain-specific behavior already observed in previous works for *A. baumannii* [8,9,10]. In addition, this observation is in agreement with our previous work, when the phenotypic behavior and transcriptomic analysis of the AB5075 was studied in the presence of HPF or CSF, respectively [8,15], showing fewer behavioral changes for the highly virulent strain.

In sum, HPF components and distinctively HSA produce strain-specific responses in all tested *A. baumannii* isolates tested. Quorum sensing, quorum quenching, fatty acids metabolism, and high-efficient iron uptake systems are differentially expressed in the presence or absence of these components. These changes are most probably adjusted to facilitate the persistence, survival, and establishment of *A. baumannii* in the different locations within the human host and fluids’ presence. The bases for the variations between strains remain to be elucidated. More detailed studies of virulence factors or other essential functions in each isolate may clarify the different adaptation requirements when HPF or HSA are present.

## Data Availability

Both RNA-seq data were deposited in the Gene Expression Omnibus (GEO) database under the accession number GSE167117.

## Supplementary Materials

The following are available online at https://www.mdpi.com/article/10.3390/pathogens10040471/s1, Table S1. Genes associated with metabolic processes with differential expression in *A. baumannii* AB5075 under HPF or HSA induction; Figure S1. Agar plate assay for the detection of AHL in *A. baumannii* AB5075 exposed to HPF, dHPF, or dHPF + HSA using *C. violaceum*. Figure S2 and Table S2. Total Iron concentration in HPF, dHPF, and dHPF + HSA conditions studied. Figure S3. Heatmap outlining the differential expression of genes associated with acinetobactin production and utilization in presence of HPF or HSA. Figure S4. Differential expression of fatty acid metabolism genes. Figure S5. Differential expression of acetoin metabolism genes. Figure S6. Differential expression of significant genes in presence of HPF or HSA. Figure S7. Heatmap outlining the differential expression of genes related to beta-lactam resistance in presence of HPF or HSA. Figure S8. SDS PAGE showing the proteins present in LB, HPF, dHPF and dHPF + HSA conditions.
