# Supplementary material for: Human Pleural Fluid and Human Serum Albumin Modulate the Behavior of a Hypervirulent and Multidrug-Resistant (MDR) Acinetobacter baumannii Representative Strain
Source: Pathogens. 2021 Apr 13;10(4):471. doi: 10.3390/pathogens10040471 (PMC8069197; doi:10.3390/pathogens10040471)
Supplement: Supplementary file 1 [file pathogens-10-00471-s001.zip › Supplementary Materials.pdf]

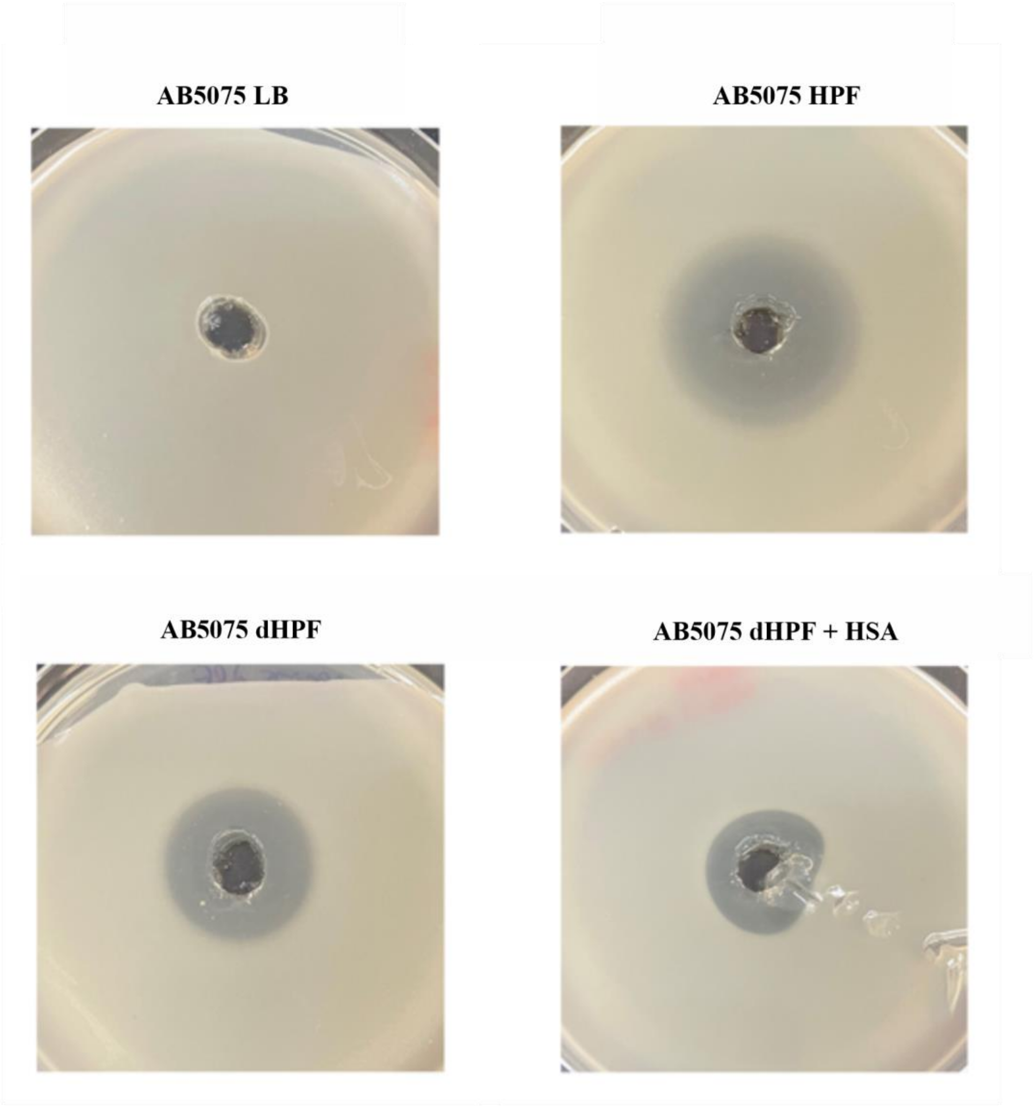

Figure S1. Agar plate assay for the detection of AHL in *A. baumannii* AB5075 exposed to HPF, dHPF, or dHPF + HSA using *C. violaceum*.

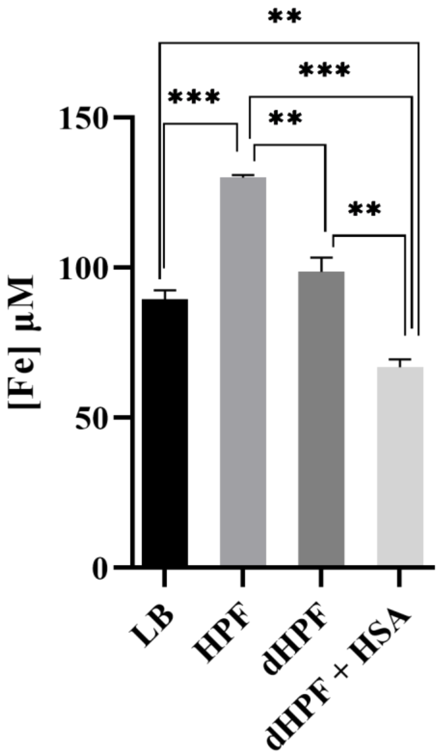

$[Fe]_{total}$ (μM)	
LB	89.38 ± 3.08
HPF	129.97 ± 0.86
dHPF	98.61 ± 4.76
dHPF + HSA	66.75 ± 2.74

Figure S2 and Table S2. Total Iron concentration in HPF, dHPF, and dHPF + HSA conditions studied.

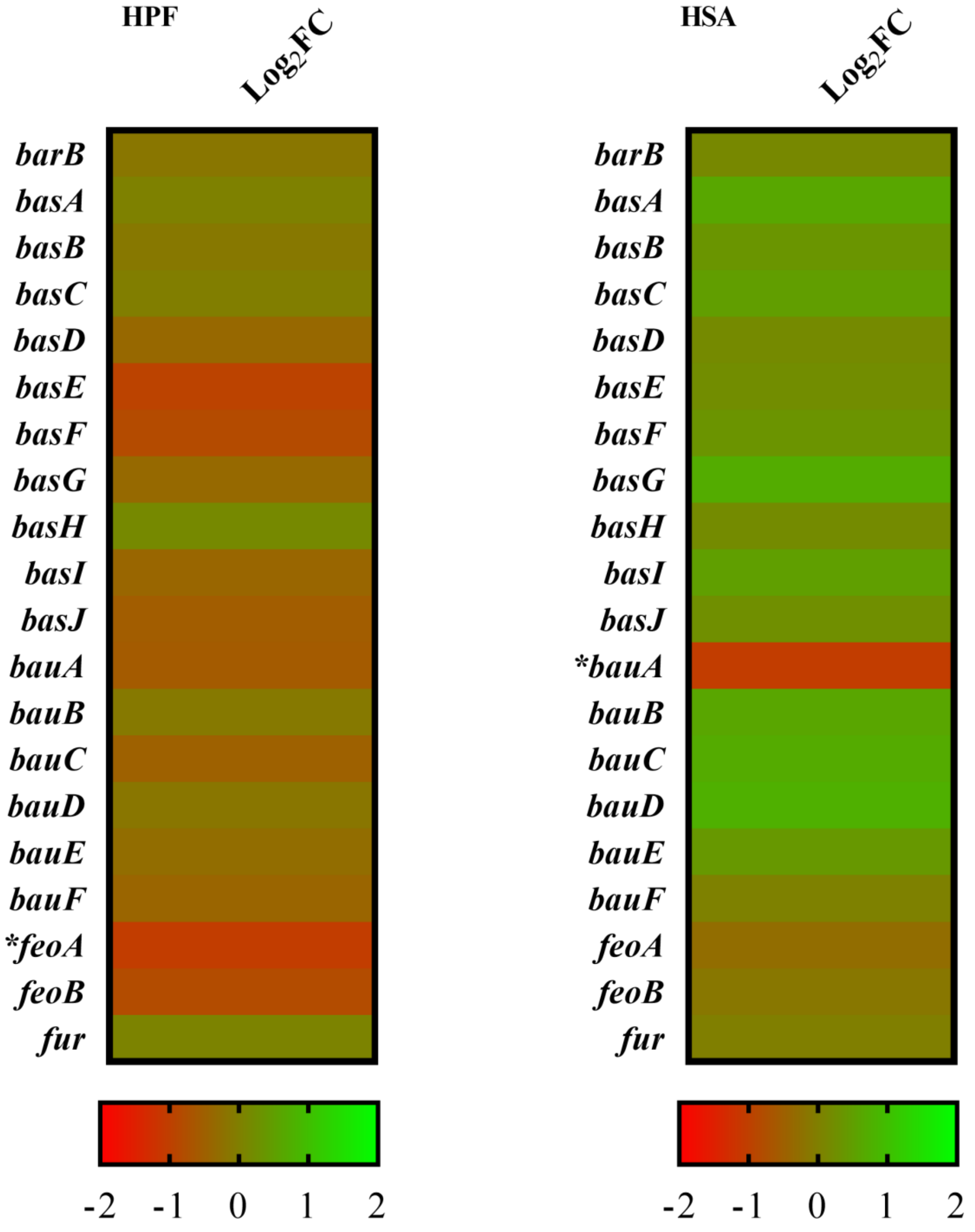

Figure S3. Heatmap outlining the differential expression of genes associated with acinetobactin production and utilization in presence of HPF or HSA. The asterisks represent significant DEGs (adjusted *P*-value < 0.05 with log2fold change > 1).

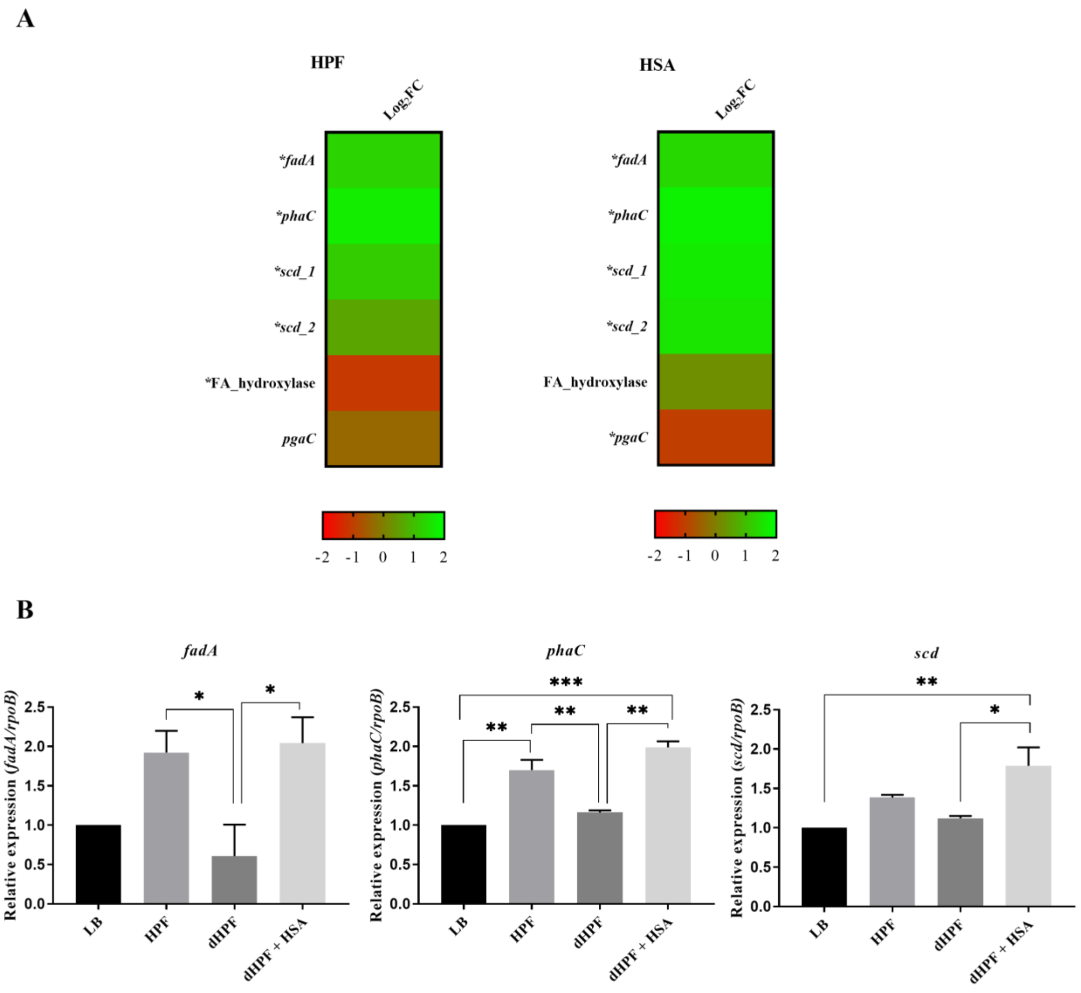

Figure S4. Differential expression of fatty acid metabolism genes. (A) Heatmap outlining the differential expression of genes associated with fatty acid metabolism in presence of HPF or HSA. The majority of fatty acid metabolism associated genes are up-regulated (green) in the presence of HPF and HSA. The asterisks represent significant DEGs (adjusted *P*-value < 0.05 with log2fold change > 1). (B) qRT-PCR of AB5075 strain genes associated with fatty acid metabolism, *fadA*, *phaC*, *scd* and *pgaC* in the presence of HPF, dHPF, or dHPF + HSA. Fold changes were calculated using double ΔCt analysis. At least three independent samples were used, and four technical replicates were performed from each sample. Statistical significance ($P < 0.05$) was determined by ANOVA followed by Tukey's multiple-comparison test.

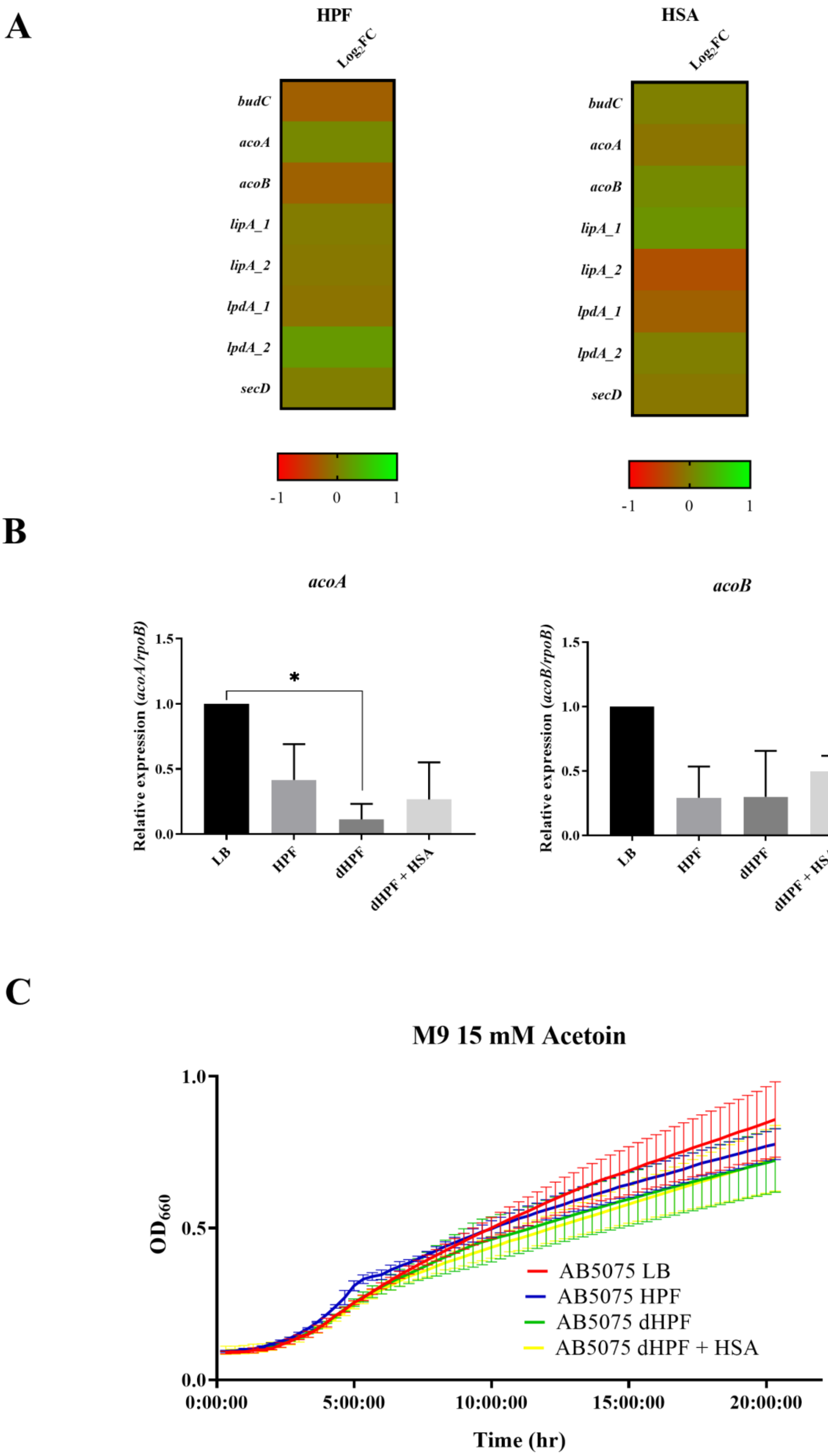
A
HPF
Log2FC
budC
acoA
acoB
lipA_1
lipA_2
lpdA_1
lpdA_2
secD
-1
0
1
HSA
Log2FC
budC
acoA
acoB
lipA_1
lipA_2
lpdA_1
lpdA_2
secD
-1
0
1
B
acoA
Relative expression (acoA/rpoB)
1.5
1.0
0.5
0.0
*
LB
HPF
dHPF
dHPF + HSA
acoB
Relative expression (acoB/rpoB)
1.5
1.0
0.5
0.0
LB
HPF
dHPF
dHPF + HSA
C
M9 15 mM Acetoin
OD660
1.0
0.5
0.0
AB5075 LB
AB5075 HPF
AB5075 dHPF
AB5075 dHPF + HSA
0:00:00
5:00:00
10:00:00
15:00:00
20:00:00
Time (hr)

Figure S5. Differential expression of acetoin metabolism genes. (A) Heatmap outlining the differential expression of genes associated with acetoin metabolism in presence of HPF or HSA. The asterisks represent significant DEGs (adjusted *P*-value < 0.05 with log2fold change > 1). (B) qRT-PCR of AB5075 strain genes associated with acetoin metabolism, *acoA* and *acoB* in the presence of HPF, dHPF, or dHPF + HSA. Fold changes were calculated using double ΔCt analysis. At least three independent samples were used, and four technical replicates were performed from each sample. Statistical significance (*P* < 0.05) was determined by ANOVA followed by Tukey's multiple-comparison test. (C) Growth curves of AB5075 strain in M9 minimal medium supplemented with 15mM acetoin at 37ºC with agitation. Strain AB5075 was grown in LB broth, HPF, dHPF, or dHPF + HSA.

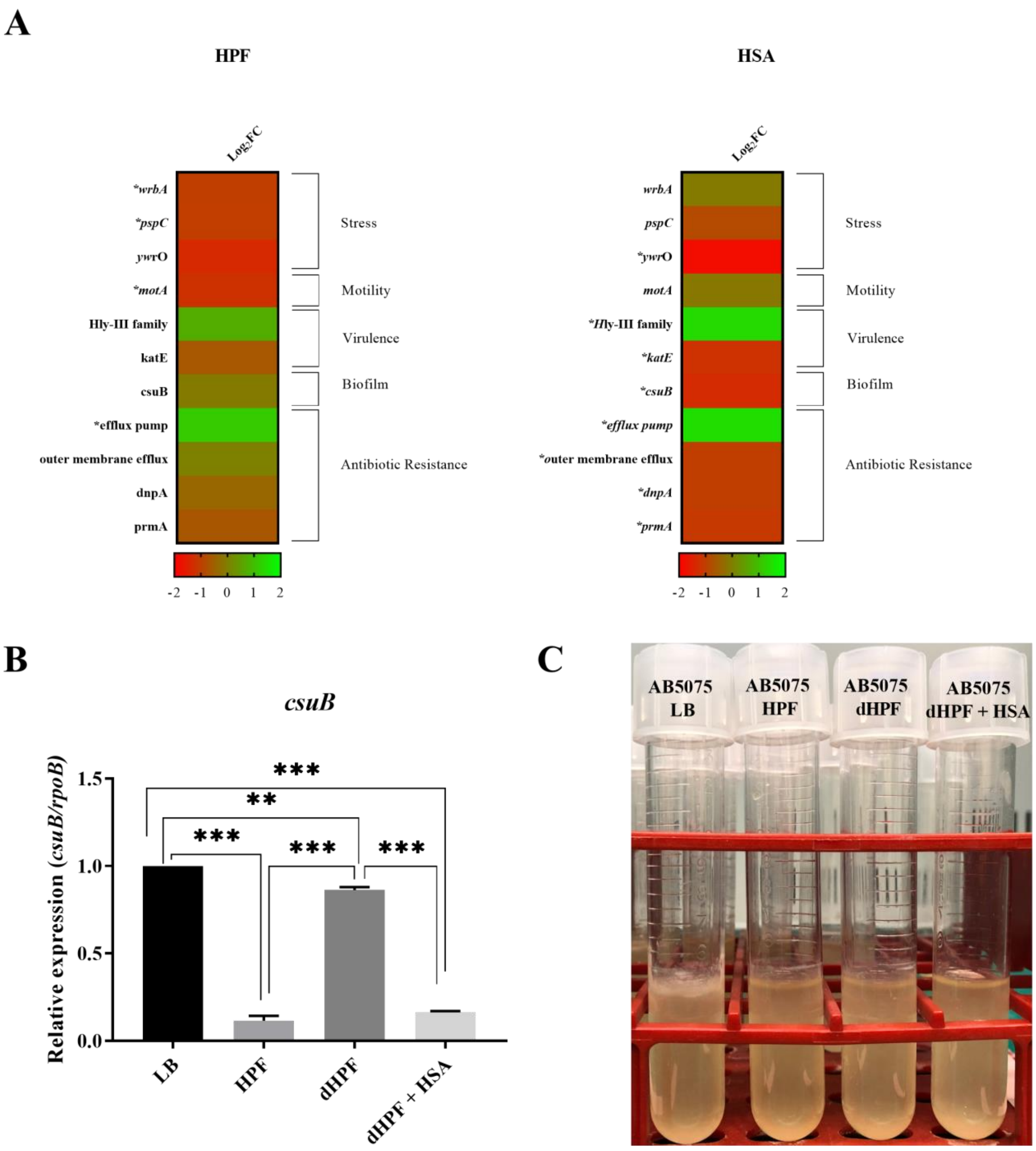

Figure S6. (A) Heatmap outlining the differential expression of significant genes in presence of HPF or HSA. The asterisks represent significant DEGs (adjusted *P*-value < 0.05 with log2fold change > 1). (B) qRT-PCR of AB5075 strain gene associated with biofilm formation, *csuB* in the presence of HPF, dHPF, or dHPF + HSA. Fold changes were calculated using double ΔCt analysis. At least three independent samples were used, and four technical replicates were performed from each sample. Statistical significance ($P < 0.05$) was determined by ANOVA followed by Tukey's multiple-comparison test. C) Biofilm assays performed in HPF, dHPF, or dHPF + HSA show a significant decrease in biofilm formation respect to LB condition.

Experiments were performed in triplicate, with at least three technical replicates per biological replicate.

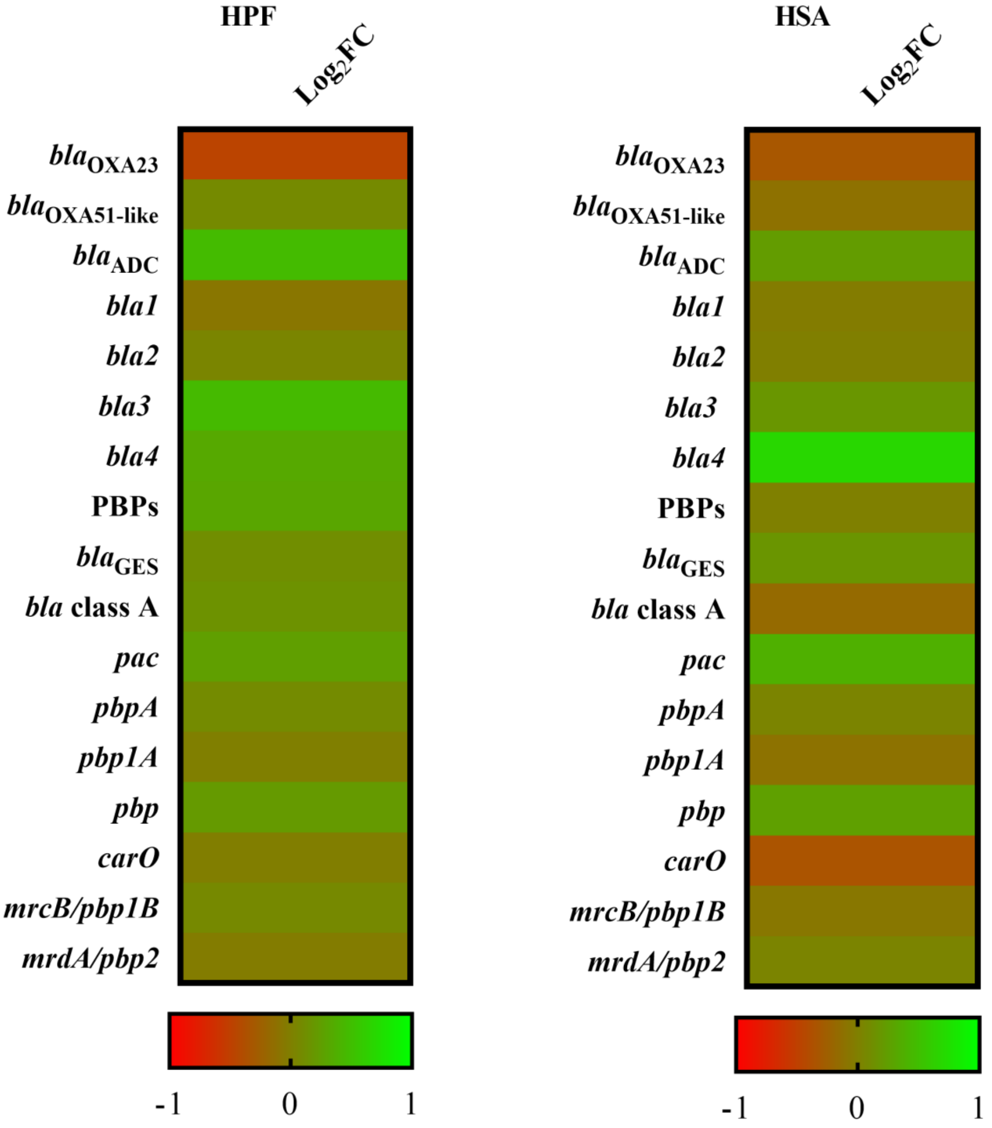

Figure S7. Heatmap outlining the differential expression of genes related to beta-lactam resistance in presence of HPF or HSA. The asterisks represent significant DEGs (adjusted *P*-value < 0.05 with log2fold change > 1).

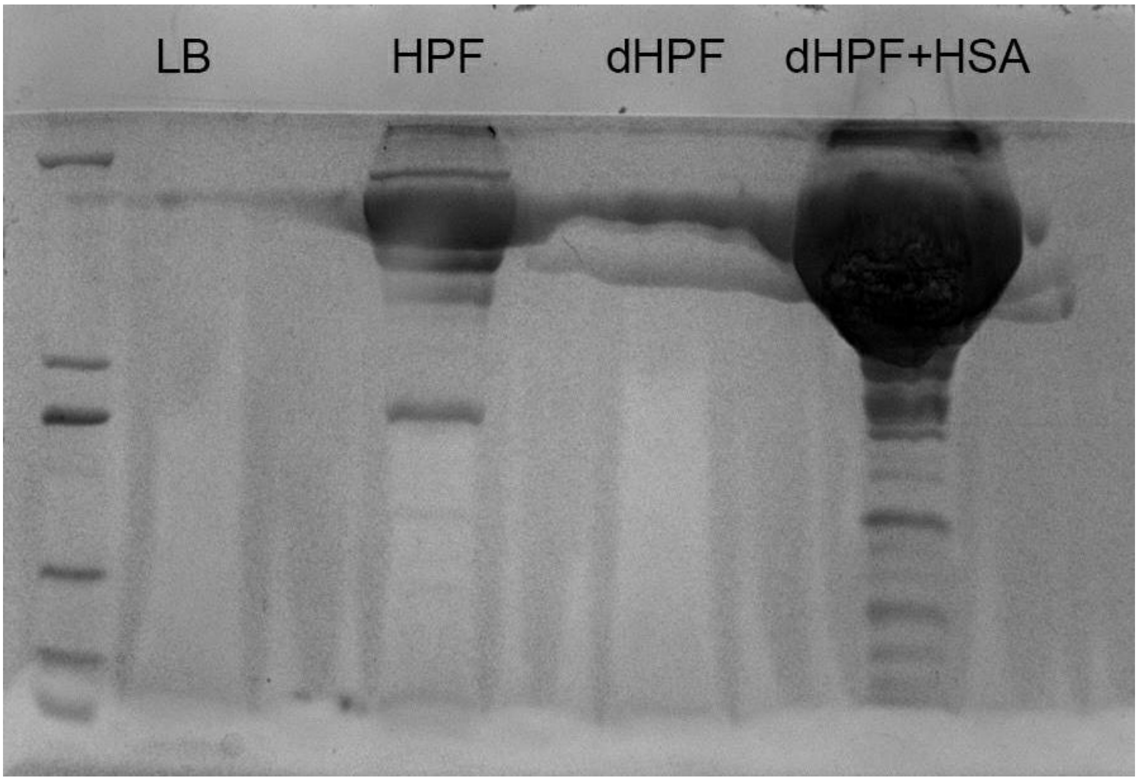

Figure S8. SDS PAGE showing the proteins present in LB, HPF, dHPF and dHPF + HSA conditions.
